# Identifying Probable Suicide Clusters in Wales Using National Mortality Data

**DOI:** 10.1371/journal.pone.0071713

**Published:** 2013-08-28

**Authors:** Phillip Jones, David Gunnell, Stephen Platt, Jonathan Scourfield, Keith Lloyd, Peter Huxley, Ann John, Babar Kamran, Claudia Wells, Michael Dennis

**Affiliations:** 1 College of Medicine, Institute of Life Sciences 2, Swansea University, Swansea, United Kingdom; 2 School of Social and Community Medicine, University of Bristol, Bristol, United Kingdom; 3 Centre for Population Health Sciences, University of Edinburgh Medical School, United Kingdom; 4 School of Social Sciences, Cardiff University, United Kingdom; 5 Office for National Statistics, Government Buildings, Newport, United Kingdom; The University of Queensland, Australia

## Abstract

**Background:**

Up to 2% of suicides in young people may occur in clusters i.e., close together in time and space. In early 2008 unprecedented attention was given by national and international news media to a suspected suicide cluster among young people living in Bridgend, Wales. This paper investigates the strength of statistical evidence for this apparent cluster, its size, and temporal and geographical limits.

**Methods and findings:**

The analysis is based on official mortality statistics for Wales for 2000–2009 provided by the UK's Office for National Statistics (ONS). Temporo-spatial analysis was performed using Space Time Permutation Scan Statistics with SaTScan v9.1 for suicide deaths aged 15 and over, with a sub-group analysis focussing on cases aged 15–34 years. These analyses were conducted for deaths coded by ONS as: (i) suicide or of undetermined intent (probable suicides) and (ii) for a combination of suicide, undetermined, and accidental poisoning and hanging (possible suicides). The temporo-spatial analysis did not identify any clusters of suicide or undetermined intent deaths (probable suicides). However, analysis of all deaths by suicide, undetermined intent, accidental poisoning and accidental hanging (possible suicides) identified a temporo-spatial cluster (p = 0.029) involving 10 deaths amongst 15–34 year olds centred on the County Borough of Bridgend for the period 27^th^ December 2007 to 19^th^ February 2008. Less than 1% of possible suicides in younger people in Wales in the ten year period were identified as being cluster-related.

**Conclusions:**

There was a possible suicide cluster in young people in Bridgend between December 2007 and February 2008. This cluster was smaller, shorter in duration, and predominantly later than the phenomenon that was reported in national and international print media. Further investigation of factors leading to the onset and termination of this series of deaths, in particular the role of the media, is required.

## Introduction

Suicide is one of the leading causes of death in young people. In Wales, the country in the United Kingdom (UK) where this study is based, suicide accounts for almost one in five deaths among men aged 15–24 and almost one in 10 deaths among women of that age [1].

A suicide cluster can be defined as an excessive number of suicides occurring in close temporal and geographical proximity [2]. A recent analysis using space-time (temporo-spatial) models over an 18 year period in New Zealand found that 1.3% of probable suicides occurred in clusters [2]. In the United States (U.S.) it has been estimated that at least 2% of teenage suicides occur in temporo-spatial clusters; clustering is thought to be two to four times more common among young people (aged 15–24 years) than among other age groups [3], [4]. Temporo-spatial analyses of specific groups of people at risk of suicide have identified ‘point clusters’: particularly in those who have contact with mental health services [5] or are in psychiatric hospitals [6]; prisons [7]; and schools [8].

Our understanding of what triggers a suicide cluster, what causes it to continue and eventually subside, is limited. Joiner [9] theorises that already vulnerable individuals, who are socially connected through shared characteristics, are those most affected by the suicide of a peer. Most researchers, however, have used the analogy of contagious illness, suggesting that there is imitation of suicidal behaviour, with social learning theory [10] being the dominant theoretical perspective. As well as local social networks, media reporting [11], and the internet [12] have been seen as important channels of transmission for suicide contagion.

In January 2008 the UK news media began reporting on a series of deaths amongst young people in South Wales, speculating that the town of Bridgend was experiencing a suicide epidemic (South Wales Echo January 17^th^ 2008; The Mirror January 23^rd^ 2008; Daily Mail, January 23^rd^ 2008). The intensity of the reporting remained high for several weeks, and the numbers of cases reported in the media continued to rise.

With the development of geospatial analysis methods, and geographic information systems in particular, it is now possible to perform more refined analyses than used in previous studies to identify suicide clusters. In this study we report the results of a Space Time Permutation Scan Statistics analysis [13] of suicides in Wales between 2000 and 2009.

The objectives of this research were to:

1. Assess the strength of statistical evidence that a group of deaths in Bridgend during 2007–2008, reported as suicide by the media, represented a temporo-spatial cluster, and if so, to identify its size, and temporal and geographical limits.
2. Identify any other temporo-spatial clusters of possible suicides across Wales during the ten year period 2000–2009.

## Methods

### Data

Data for all deaths classified as suicide, undetermined intent, and accidental for residents of Wales between 1st January 2000 and 31st December 2009 were supplied by the Office for National Statistics, Newport. Information on each case included: date of death, age at time of death, sex, International Classification of Disease (ICD) code for cause of death, and 12-figure Ordinance Survey (OS) geographic grid reference of place of residence at the time of death.

Traditionally, official suicide statistics in the UK combine deaths that have received a coroner's verdict of suicide (intentional self-harm) as well as those with undetermined intent (open verdicts), as most such deaths are suicides [14]. These deaths are coded by the Office for National Statistics (ONS) in England and Wales using the ICD tenth revision (ICD-10) codes X60–84 (intentional self-harm) and Y10–34 (death of undetermined intent). Our primary analysis was based on such deaths, excluding those coded Y33.9/U50.9 (pending verdicts) as a large proportion of these are subsequently found to be homicides [15].

In recent years there has been an increase in the number of ‘narrative verdicts’ recorded by coroners in England and Wales. In a narrative verdict the conclusions of the coroner are recorded as a brief summary, usually several sentences describing the circumstances of the death [16], [17]. The Office for National Statistics (ONS), code deaths given narrative verdicts on the basis of information on intent noted by the coroner. If no intent is specified in deaths from injury or poisoning then they are coded as accidents [16], [17]. The increased use of narrative verdicts could thereby potentially increase the official rate of accidental deaths at the expense of those classified as intentional self-harm or undetermined intent, leading to an underestimate of probable suicide [16], [18]. Furthermore, there is evidence that a high proportion of deaths from poisoning and hanging that receive accidental verdicts, are found, when subjected to clinical review, to be suicides [19]. For this reason we carried out secondary analyses that also included deaths coded as accidental hanging and strangulation (W75 & 76) and accidental poisoning from substances other than narcotics and psychodysleptics/hallucinogens (X40 & 41, 43–49). Accidental deaths from poisoning by narcotics and psychodysleptics/hallucinogens (X42) were excluded as these are common drugs of misuse, and intent may be particularly difficult to determine for such deaths.

### Analysis

The age and sex distribution of probable and possible suicides in Wales from 2000 to 2009 were described graphically and chi square statistics were used to compare proportions. Rates of probable and possible suicide were calculated using mid-year population estimates [20].

Space Time Permutation Scan Statistics (STPSS) were used to test for the presence of temporo-spatial clusters across Wales for the period 1st January 2000 to 31st December 2009 [13]. Monte Carlo ranking is used to assess the strength of statistical evidence (*p*-value) for the occurrence of clustering. The analysis was carried out in SaTScan v9.1 [21]. In order to run a temporo-spatial analysis SaTScan needs both the time and geo-location for each event. Time of event was recorded as the day of death and the place of residence as a 12 figure Ordinance Survey grid reference, pinpointing the location of each case to the nearest metre. STPSS investigates clustering within a variable time window across varying geographical areas and uses a Poisson based likelihood to compare the expected number of cases and actual number of cases inside and outside the window. The size of the time and geographical window can be varied within pre-specified limits. The result is a set of cylinders where the base represents the area of the potential cluster and the height represents the time period of the cluster.

The temporo-spatial window with the biggest likelihood ratio of cases is reported as the most likely candidate for a temporo-spatial cluster. The variation in the size of the scanning window is specified as a percentage of the map area and as a percentage of the time duration in the data. For our analysis the variable scan window was set to a maximum of 10% of the study area (approximately 50 km radius) and 10% of the study period (equal to 1 year). A systematic review of all published studies reporting suicide clusters between 1977 and 2009 [2] has indicated a median duration of 11 months for narrative reports of clusters, further justifying a 12 months scan window. The shape of the spatial scan window can be circular or elliptical. When an elliptical shaped window is selected, the scanning window varies in shape as well as in size, and is repeated for different rotations of the ellipse. The elliptical shape changes from longer and narrower towards circular, thereby reducing the likelihood of missing clusters that are associated with linear settlements, or along particular streets within larger settlements. For the analysis of clusters the system was set to allow geographic overlap between potential clusters, so that two or more clusters could include the same geographical space, provided that the centre of the less likely cluster was not within the area of a more likely cluster. This allows for the detection of neighbouring clusters that might share some of the cases. The model also adjusts for clustering in space and time alone, thereby adjusting for areas and seasons with a higher baseline rate.

Temporo-spatial cluster analysis was performed on four pre-specified groups of deaths:

1. Probable suicides amongst people aged 15 years and over: intentional self-harm (ICD-10 codes X60–84), and deaths of undetermined intent (ICD-10 Y10–34 excluding Y33.9);
2. Probable suicide amongst people aged 15 to 34 years: intentional self-harm (X60–84), and deaths of undetermined intent (Y10–34 excluding Y33.9);
3. Possible suicide for people aged 15 years and over: probable suicides (see above) and accidental poisoning (X40,41,43–49) and strangulation (W75,76) deaths;
4. Possible suicide for people aged 15 to 34 years: probable suicides (see above) and accidental poisoning (X40,41,43–49) and strangulation deaths (W75,76).

Our focus on 15–34 year olds reflected the research evidence [3], [4] that clusters occur more frequently in young people and the particular concern that the ‘cluster’ in Bridgend had occurred amongst the young.

### Ethical approval

This study is a component of the study entitled ‘Using routinely collected data from suicide clusters to influence social and health care service delivery: an investigation of the Bridgend cluster’ that received full ethical approval from the South West Wales Research Ethics Committee (REC reference: 10/WMW02/10).

## Results

There were 3943 deaths included in the analysis: 3002 (76.1%) males and 941 (23.9%) females. Altogether 2375 (60.2%) of the deaths were coded as suicide (intentional self-harm), 616 (15.6%) undetermined and 952 (24.1%) accidental poisoning (excluding narcotics) or accidental hanging (Table 1).

**Table 1:** Cause of death groups according to ICD codes of possible suicides for Wales 2000–2009.

	All Wales 15+ n (%)	All Wales 15–34 n (%)	Bridgend 15+ n (%)	Bridgend 15–34 n (%)
Intentional self-harm (ICD X60–84)	2375 (60)	720 (56)	117(57)	55 (59)
Undetermined intent (ICD Y10–34, excluding Y33.9)	616 (16)	194 (15)	32(16)	12 (13)
Accidental (ICD W75/76 & X40,41, 43–49)	952 (24)	375 (29)	55(27)	26 (28)
*Accidental poisoning (X40,41, 43–49)*	*833 (87.5)*	*318 (85)*	*41(74.5)*	*18 (69)*
*Accidental hanging and strangulation (W75/76)*	*119 (12.5)*	*57 (15)*	*14(25.5)*	*8 (31)*
**TOTAL (%male)**	**3943 (76%)**	**1289 (83%)**	**204 (80%)**	**93 (85%)**

**Figure 1:**
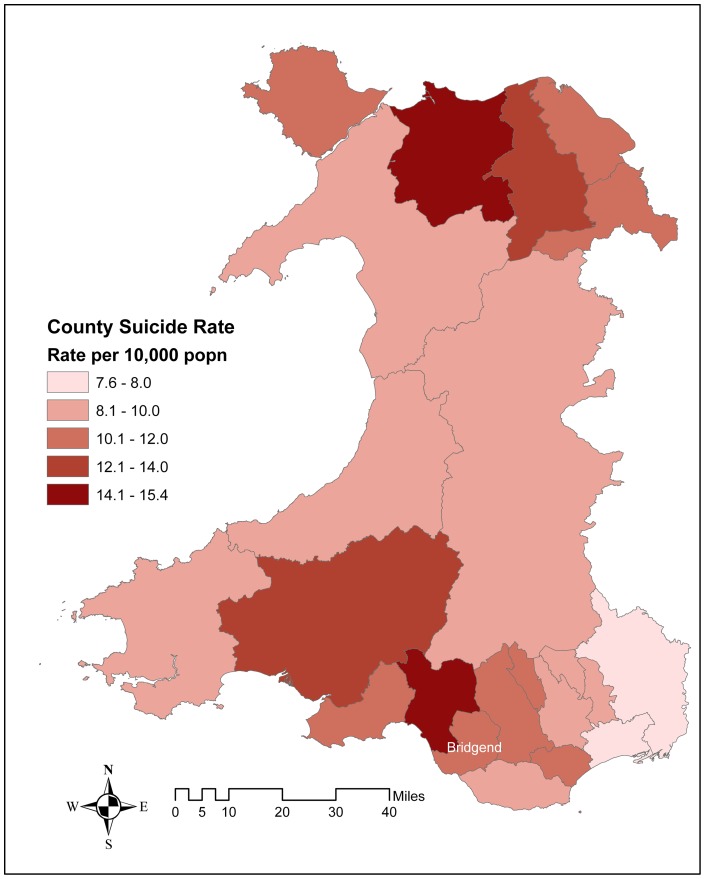
Ten year rates of probable suicides (suicides and undetermined deaths) for Welsh Counties 2000–2009. The geographical variations in rates of probable suicide across Welsh counties 2000 to 2009.

Figure 1 shows the geographical variations in rates of probable suicide across Welsh counties. Rates of probable suicide in Bridgend (11.4/100,000/year, 95% CI 8.7–14.1) were only marginally higher than those for the whole of Wales (10.2/100,000/year, 95% CI 9.3–11.0). This was also the case for possible suicide (suicide, undetermined, and accidental poisoning and hanging) with a mean Bridgend rate of 15.6/100,000/year (95% CI 13.4–17.7) compared to 13.4 /100,000/year (95% CI 12.6–14.1) for the whole of Wales. The sex and cause of death distribution of deaths were similar in Bridgend to those seen for all Wales (Table 1), with the exception that in both all ages combined and 15–34 year olds there was statistical evidence (*p* = 0.002 and *p* = 0.019, respectively) of a higher proportion of accidental hanging/strangulation compared to accidental poisoning deaths in Bridgend than the rest of Wales.

### Rate of death by cause across time for Wales

**Figure 2:**
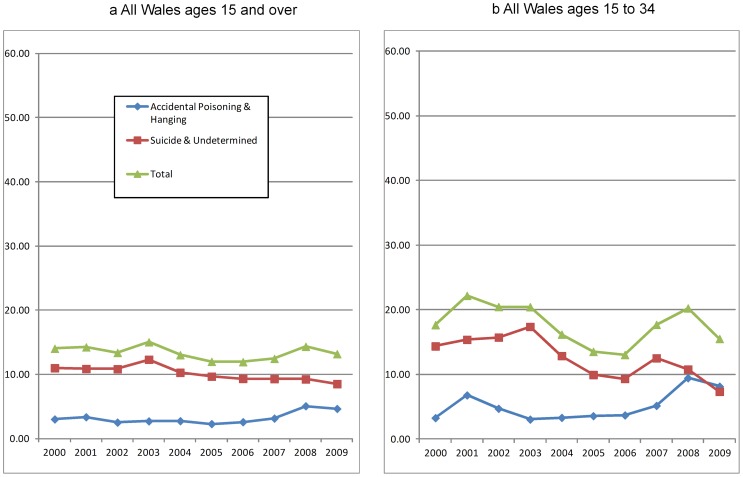
Mortality statistics for Wales by cause of death group (rate per 100,000 population). Apart from a small rise in 2003, deaths coded as suicide and of undetermined intent decreased between 2000 and 2009 in Wales as a whole (Figure 2a). Deaths coded as accidental poisoning and hanging decreased slightly from 2000 to 2005, before starting to increase in 2006 and rising by 61% in 2008. For possible suicides there were two peaks in the period: the first in 2003, which was due to a rise in deaths coded as suicide and of undetermined intent; and the second in 2008, which was due to a rise in deaths coded as accidental poisoning or accidental hanging. For ages 15 to 34 years there is a similar pattern but with greater variation (Figure 2b).

Apart from a small rise in 2003, deaths coded as suicide and of undetermined intent decreased between 2000 and 2009 in Wales as a whole (Figure 2a). Deaths coded as accidental poisoning and hanging decreased slightly from 2000 to 2005, before starting to increase in 2006 and rising by 61% in 2008. For possible suicides there were two peaks in the period: the first in 2003, which was due to a rise in deaths coded as suicide and of undetermined intent; and the second in 2008, which was due to a rise in deaths coded as accidental poisoning or accidental hanging. For ages 15 to 34 years there is a similar pattern but with greater variation (Figure 2b).

### Rate of death by cause across time for the County Borough of Bridgend

**Figure 3:**
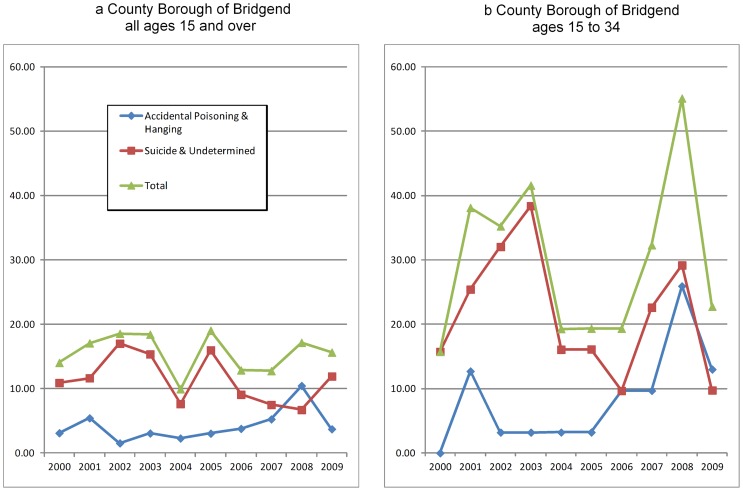
Mortality statistics for the County Borough of Bridgend by cause of death group (rate per 100,000 population). The rate of death by cause across time for the County Borough of Bridgend (Figure 3a) was considerably more varied than the rate of death by cause across Wales, as might be expected considering the relatively small population of Bridgend. In the all-age rates (figure 3a) there was an indication of a peak in accidental deaths by hanging and poisoning in 2008 and some evidence that 15–34 year olds contributed disproportionately to this excess (figure 3b). In 15–34 year olds, possible suicides peaked in 2008 (figure 3b) with a second smaller peak in 2003.

The rate of death by cause across time for the County Borough of Bridgend (Figure 3a) was considerably more varied than the rate of death by cause across Wales, as might be expected considering the relatively small population of Bridgend which varied from 128,224 in 2000 to 134,197 in 2009. The number of possible suicides in Bridgend ranged from 13 to 25 per year for all ages and 5 to 17 per year in 15–34 year olds. In the all-age rates (figure 3a) there was an indication of a peak in accidental deaths by hanging and poisoning in 2008 and some evidence that 15–34 year olds contributed disproportionately to this excess (figure 3b). In 15–34 year olds, possible suicides peaked in 2008 (figure 3b−17 deaths) with a second smallerpeak of 13 deaths in 2003.

### Space Time Suicide Clusters

**Figure 4:**
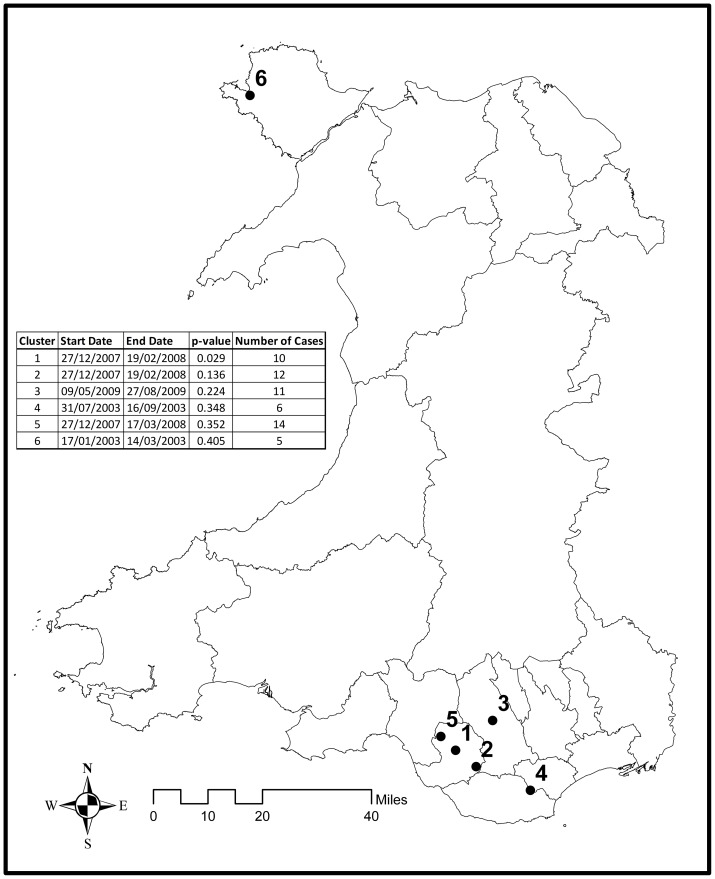
Temporo-spatial clusters of possible suicides (suicide, undetermined, and accidental poisoning and hanging) for people 15–34 years. The temporo-spatial analysis identified several possible clusters across Wales for age group 15 to 34 years. Three of the clusters were centred on the county borough of Bridgend, but only cluster number one was statistically significant (p = 0.029).

**Figure 5:**
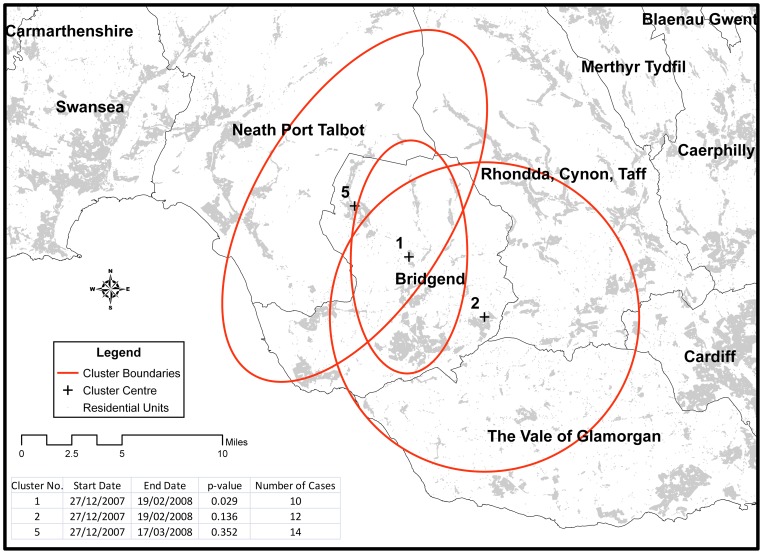
Most likely temporo-spatial clusters of possible suicide in Bridgend and surrounding boroughs 2000–2009. The three most likely clusters for the age group 15 to 34 years centred on the county borough of Bridgend. The clusters all extended into neighbouring boroughs.

When analysing deaths aged 15 and over, or when examining younger people only (aged 15–34 years), there was no statistical evidence of temporo-spatial clusters representing probable suicide (suicide and undetermined) in Wales during the period 2000–2009– the lowest *p* value being 0.48. When deaths from accidental poisoning and hanging were included (i.e. possible suicide; Figure 4), statistical evidence for a cluster was identified in the sub-set of data for people aged 15–34 year (*p* = 0.029) (see insert, Figure 4). This cluster centred on the County Borough of Bridgend but extended into the neighbouring County of Neath Port Talbot for the period 27/12/2007 to 19/2/2008. It contained 10 of the 28 suicides in 15–34 year olds in Wales during that time period. Other clusters of possible suicides in people aged 15–34 are displayed in Figure 4, but statistical evidence for these was weak (*p*>0.13). Two of these (clusters 2 and 5) occurred at similar time periods to the primary cluster and included cases from the primary cluster, but either related to a larger geographical area or were of longer duration (Figure 5). Combining the primary and secondary cases in this area for the period 27^th^ December 2007 to 19^th^ February 2008 extends the size of the cluster to a possible 15 cases.

The ten deaths in the primary cluster comprised five coded as suicide, two undetermined and three as accidental. There were another 18 deaths in the same age group elsewhere in Wales during the period of the identified cluster. The mean age of the cluster cases was 21.4 (SD 5.6) years compared to 25.7 (SD 5.8) years among non-cluster members (two-tailed t-test  = −1.884; *p* = 0.071). There was no statistical evidence of difference between cases in and outside the cluster in respect of gender (Male 60% inside, 83% outside; Female 40% inside, 27% outside: Fisher Exact test *p* = 0.21), cause of death (70% suicide inside, 61% outside; Accidental 30% inside, 39% outside: Fisher Exact test *p* = 0.71), or method of suicide (80% hanging inside, 61% hanging outside: Fisher Exact test *p* = 0.42).

The 10 cluster deaths comprised 0.78% of possible suicide deaths in 15–34 year olds over the 10 year study period and 0.25% of total (all age) suicides.

## Discussion

### Main Findings

We found statistical evidence that a cluster of 10 deaths amongst 15–34 year olds occurred in the Bridgend area of South Wales between 27^th^ December 2007 and 19^th^ February 2008 when using a broad definition of suicide. This cluster was smaller and shorter in duration than the phenomenon reported in the print media. Additionally, most deaths in the cluster occured after the commencement of the attention from the print media; much of the initial newspaper focus related to deaths in the preceeding twelve months. There was some weak statistical evidence that the cluster may have included up to 15 cases during that time. Overall only 0.78% of possible suicides in 15–34 year old Wales (0.25% of total all-age suicides) over the ten year period were identified as being cluster-related. The number of cluster related deaths may, however, be an under-estimate as it is possible that small clusters go undetected using Space Time Permutation Scan Statistics.

The analysis has highlighted several important issues in relation to the analysis of clusters of suicide. First, in this study the cluster of suicides was only detected when the analysis was restricted to cases aged 15 to 34 years. This age group accounts for only a third of all suicides and without such a sub-group analysis the possible cluster might have been overlooked. Investigation of this group was pre-specified in view of both the reported characteristics of deaths associated with the cluster and previous literature suggesting that this age group may be particularly susceptible to the factors that influence cluster development [4].

Second, as a result of increases in the use of narrative verdicts by coroners and the consequent possible coding of some probable suicides as accidental deaths by ONS [16], [17] as well as the use of accidental verdicts for some likely suicides [14], [22], the cluster was only detected by applying a broader definition of suicide than usually employed in epidemiological studies of suicide. In England the number of narrative verdicts has doubled in 5 years, and in Wales this has tripled [23]. Due to variation in coroners' use of narrative verdicts, regional and local effects may be more pronounced [18]. Any bias introduced by the use of narrative verdicts becomes particularly important when considering the possibility of the presence of a point cluster. Furthermore, there is increasing evidence that many deaths recorded as accidental poisoning and hanging represent probable suicide [19]. Additionally, risk factors associated with accidental death from non-narcotic poisoning as well as other causes of accidental death are similar to risk factors for deaths recorded as suicide [24].

### Comparisons with other studies

The proportion of possible suicides in younger people identified in this study occurring as part of a cluster (0.78%) is lower than previously reported in the literature. Larkin & Beautrais [2], in their analysis of suicide clusters in New Zealand during 1990–2007, found that 1.3% of probable suicides for all ages occurred in clusters. Other studies of the temporo-spatial clustering of suicides have used the Knox method [3], [4] and shown that at least 2% of teenage suicides in the U.S. occur in clusters; however there are a number of limitations to this method [25]. Additionally, cross-national comparisons need to be reviewed cautiously because of a variety of factors affecting cause of death recording [26].

### Strengths and limitations of this study

There are two main strengths of the study. The first is the use of point data giving the precise location of residence for the suicide deaths, thereby avoiding the need for calculating rates for artificially defined areas [27]. The second is our use of state of the art methods for assessing clustering – Space Time Permutation Scan Statistics [13]. The principal limitation of the study is the reliability of official suicide statistics (see above). We have attempted to minimise problems relating to the use of narrative verdicts and ONS coding by performing an analysis based on probable suicide (suicide and undetermined deaths) and possible suicide (suicide, undetermined, and accidental hanging and poisoning deaths, excluding narcotic-related mortality). Inevitably there is the possibility that some accidental deaths are not due to suicidal behaviour, and this is also true of a proportion of deaths receiving an open verdict [14].

Analysis of clusters such as this assumes that all relevant suicides occur within relatively close geographic proximity. Given the ease of communication and travel in the 21^st^ century it is possible that suicides amongst those who are part of an affected friendship group may live some distance outside the geographic areas used in our scanning methodology and so not be identified as members of the cluster. Such cases may only be identifiable through local knowledge and discussion with an affected community. Additionally, approaches such as those used here may lack power to delineate all cluster-related suicides – for example point clusters in institutions when only two or three deaths occur within a single school, particularly where catchment areas for school are dispersed.

Establishing the presence of a putative suicide cluster through sophisticated analysis and geographical mapping may be of restricted value. Firstly, the limitations previously outlined, as well as the late availability and reliability of data, are a major consideration. Secondly, community perception is much more immediately relevant in implementing an appropriate response strategy [28]. Statistical evidence for the Bridgend cluster is relatively weak, and at the early stages of the cluster local health authorities were not convinced that the number of suicides occurring was above that expected given normal fluctuations in rare events. In contrast, there was strong concern within the community that an excess of suicides was occurring. Additionally, the local perception of clustering and consequential effects on the community may increase the contagious effects of suicide [28]. The presence of a pre-existing community strategy may be particularly beneficial for communities in early identification of the risk of a cluster developing, supporting vulnerable individuals, and managing relationships with the media [29], [30].

### Conclusions and implications for future research

We have found evidence for the occurrence of a temporo-spatial cluster of possible suicide centred on the County Borough of Bridgend and overlapping the time period of the early media reporting of a cluster. It represented only 0.78% of possible suicides in young people in Wales over a ten year period. Factors relating to the initiation, maintenance and cessation of this suicide cluster, as with others remain unclear. A recent systematic review examining suicide cluster risk factors and mechanisms identified some important associations, particularly direct involvement with another cluster member, and other factors known to be linked to suicide generally such as a history of self harm and substance misuse [31]. Although contagion was the most prevalent mechanism of cluster formation reported, other mechanisms such as homophily (people preferentially assorting to similar others) may also be operating independently or in combination. One important avenue for research could be interviewing individuals who survived a serious attempt at suicide and were part of a cluster of suicidal behaviour [31]. There is unequivocal evidence from systematic reviews that media reporting of suicide may be associated with further suicides particularly using the same method [32], [33], [34], [35]; this is particularly relevant to ‘mass’ clusters when there is a temporary increase in the total frequency of suicides for a population relative to the time preceding and after the cluster. The role of the print media in temporo-spatial clusters, however, has not been investigated. A number of important observations suggest that the effects of media reporting for the cluster we have detected requires further scrutiny. Firstly, the cluster was predominantly later, smaller and shorter in duration than the phenomenon that was widely reported in national and international print media. Secondly there was a high profile of reporting at the time, and thirdly there were no other clusters in Wales during the ten year period, as well as no evidence of previous clusters in the same area that would indicate any specific community vulnerability.
